# 

**Published:** 1896-06

**Authors:** 


### CONTENTS FOR JUNE, 1896.

ORIGINAL COMMUNICATIONS. PAGE.

A Case of Retroflexion of the Gravid Uterus. By Dr. Gabriele Baronin Possanner.... 849
College Life for Women Twenty Years Ago. By Mary Blair-Moody, M. D.............. 854
Some Lesions of the Pelvic Viscera not Requiring Operative Treatment. By Bina Potter-Van Denbergh, M. D........ 859
A Case of Extensive Cerebral Softening. By Helene Kuhlmann, M. D. ........ 864
Schauta's Clinic—Some Impressions of Interest in Obstetrics and Gynecology. By Electa B. Whipple, A. M., M. D.......... 866
Cystic Urachus. By Jane W. Carroll, M. D........ 869
Riverside Hospital. By Lillian Craig Randall, M. D..... 872
Hypertrophy of the Lingual Tonsil. By Mary E. Dickinson, M. D.......... 878

CLINICAL REPORT.

Three Cases of Deformed Fetal Skull.......... 880

TRANSLATION.

Bacteriological Study of 117 Cases of Scarlatina Angina.......... 883

SOCIETY PROCEEDINGS.

Buffalo Academy of Medicine .......... 886

PROGRESS IN MEDICAL SCIENCE.

Surgery.......... 889
Obstetrics, Gynecology and Pelvic Surgery .......... 893
Pediatrics.......... 896
Public Health and School Sanitation.......... 898

EDITORIAL.

College Commencements and Alumni Association Meetings.......... 901
Woman and the Bicycle .......... 908
The Erie County Hospital .......... 909
An Object Lesson for Antivaccinists.......... 909
A Remarkable Woman in Medicine .......... 910
Topics of the Month .......... 912

Notes About Women.......... 916
Personal.......... 916
Obituary.......... 917
Hospital Note.......... 918
Society Meetings.......... 918
Removals .......... 921
Reviews.......... 922
Literary Notes.......... 922
Miscellany .......... 928

